# Prevalence and effects of multiple chemical sensitivities in Australia

**DOI:** 10.1016/j.pmedr.2018.03.007

**Published:** 2018-03-10

**Authors:** Anne Steinemann

**Affiliations:** Department of Infrastructure Engineering, Melbourne School of Engineering, The University of Melbourne, Melbourne, Victoria 3010, Australia; College of Science, Technology and Engineering, James Cook University, Townsville, Queensland 4811, Australia; Climate, Atmospheric Sciences, and Physical Oceanography, Scripps Institution of Oceanography, University of California, La Jolla, San Diego, CA 92093, USA

**Keywords:** MCS, Multiple chemical sensitivities, Chemical sensitivity, Asthma, Fragrance sensitivity, Fragranced consumer products

## Abstract

Multiple chemical sensitivities (MCS) is a medical condition associated with exposure to common chemical pollutants. The aims of this study are to assess the prevalence of MCS, its overlaps with asthma and fragrance sensitivity, and its health and societal effects in Australia. Data were collected in June 2016 using an on-line survey with a representative national sample (N = 1098) of adults (ages 18–65) in Australia. Results found that, across the country, 6.5% report medically diagnosed MCS, 18.9% report chemical sensitivity (being unusually sensitive to everyday chemicals and chemically formulated products), and 19.9% either or both. Among people with MCS, 74.6% also have diagnosed asthma or an asthma-like condition, and 91.5% have fragrance sensitivity, reporting health problems (such as migraine headaches) when exposed to fragranced consumer products (such as air fresheners and cleaning supplies). In addition, among people with MCS, 77.5% are prevented from access to places because of fragranced products, 52.1% lost workdays or a job in the past year due to fragranced product exposure in the workplace, and 55.4% report health effects considered potentially disabling. Results indicate that MCS is a widespread disease, affecting an estimated 1 million adult Australians, with chemical sensitivity affecting another 2 million. Reducing chemical exposure to problematic sources, such as fragranced consumer products, is critical to reduce adverse effects.

## 1 Introduction

People with multiple chemical sensitivities (MCS) experience adverse health effects from exposure to common chemical pollutants, often at low levels, from products such as pesticides, new carpet and paint, renovation materials, diesel exhaust, cleaning supplies, scented laundry products, air fresheners, and perfume (Ashford and Miller, 1998). Risks from exposure include a range of acute, chronic, and potentially disabling health effects, including headaches, dizziness, seizures, heart arrhythmia, gastrointestinal problems, breathing difficulties, and asthma attacks (Steinemann, 2018; Ashford and Miller, 1998). Prior studies found that people with MCS generally report a higher incidence of fragrance sensitivity (adverse health effects from exposure to fragranced consumer products) and of asthma (Steinemann, 2018; Caress and Steinemann, 2009b).

While multiple chemical sensitivities (or sensitivity) is arguably the most common term, the condition is also known as environmental illness (specific to chemical exposures), the petrochemical problem, or toxicant induced loss of tolerance (Ashford and Miller, 1998; Miller and Prihoda, 1999). Further, while progress has been made on elucidating mechanisms of disease and biomarkers (e.g., Genuis, 2010; De Luca et al., 2011; Belpomme et al., 2015), MCS still lacks a single international case definition (MCS 1999, 1999; Ashford and Miller, 1998). People with MCS may not receive a distinct medical diagnosis, but nonetheless manifest the condition of chemical sensitivity.

A hallmark of MCS is that it is typically both initiated and triggered by chemical exposures. Sources commonly implicated in MCS (e.g., pesticides, solvents, new building materials, and fragranced consumer products) are documented sources of air pollutants (McDonald et al., 2018; Ott et al., 2007). People with MCS have been likened to human canaries: they react earlier and more severely to pollutants, and at levels far lower than the general population (Ashford and Miller, 1998).

Little is known about the prevalence of MCS in Australia. In one prior study, a population-based survey of 4009 adults in South Australia in 2001–2002 found a prevalence of 15.9% of self-reported chemical sensitivity and 1% medically diagnosed MCS (Fitzgerald, 2008). Chemical sensitivity was investigated with the questions: “Is your health seriously affected by exposure to any of the following (perfume, traffic pollution, household chemicals, workplace chemicals)?” and “Do you consider yourself especially sensitive to everyday chemicals found in household cleaning products, perfumes, insect sprays, new carpets, fresh paints, etc.?”.

In other countries, a recent national study in the US (Steinemann, 2018) found a prevalence of 25.9% self-reported chemical sensitivity and 12.8% medically diagnosed MCS. Prior US national prevalence studies, conducted in 2002–2003 and 2005–2006 (Caress and Steinemann, 2005, Caress and Steinemann, 2009a), found respectively 11.1% and 11.6% self-reported chemical sensitivity and 2.5% and 3.9% medically diagnosed MCS. Thus, over the past decade, MCS prevalence has increased over 300% and chemical sensitivity over 200% in the US (Steinemann, 2018). Chemical sensitivity was investigated in these three national studies using the question: “Compared to other people, do you consider yourself allergic or unusually sensitive to everyday chemicals like those in household cleaning products, paints, perfumes, detergents, insect spray and things like that?” The fundamental question was developed by the California Department of Health Services and used in their survey of 4046 Californians (Kreutzer et al., 1999), and a modified version was also used in the survey of South Australians (Fitzgerald, 2008). Using the Quick Environmental Exposure and Sensitivity Inventory (QEESI) criteria (Miller and Prihoda, 1999), a survey of 7245 adults in Japan (Azuma et al., 2015) and 2000 adults in Denmark (Skovbjerg et al., 2012) estimated a prevalence of 7.5% and 8.2% respectively of chemical intolerance.

The aims of this present study are three-fold: (1) to determine the prevalence of medically diagnosed MCS and chemical sensitivity in Australia, (2) to investigate its co-occurrence with asthma or an asthma-like condition, and with fragrance sensitivity, and (3) to assess the impact of exposure to fragranced consumer products on health and quality of life for people with MCS. Results from this study point to ways to reduce adverse effects and promote public health.

## 2 Methods

To assess the national prevalence and effects of MCS, an on-line survey was conducted with a random sample of the Australian population, representative of age, gender, and region (N = 1098, 95% confidence level, 3% margin of error), drawn from a large national panel (over 200,000 people) held by Survey Sampling International. The survey instrument was developed and tested over a two-year period before full implementation in June 2016. Response rate was 93%, and all responses were anonymous. (For details, see “Survey Methods” and “Survey Data 2” as supplementary material.)

To promote comparability and consistency, the survey employed questions from previous national and large-scale regional MCS prevalence studies (Steinemann, 2018; Caress and Steinemann, 2004, Caress and Steinemann, 2005, Caress and Steinemann, 2009a; Fitzgerald, 2008; Kreutzer et al., 1999). For medically diagnosed MCS, the survey asked, “Has a doctor or health care professional ever told you that you have multiple chemical sensitivities?” For chemical sensitivity, the survey asked, “Compared to other people, do you consider yourself allergic or unusually sensitive to everyday chemicals like those in household cleaning products, paints, perfumes, detergents, insect spray and things like that?”.

For asthma, the survey asked, “Has a doctor or health care professional ever told you that you have asthma or an asthma-like condition?” and then further asked to specify whether asthma or an asthma-like condition. (The term “asthmatic” will be used herein to include individuals with either asthma or an asthma-like condition or both.)

For fragrance sensitivity, the survey asked about exposure to different types of fragranced consumer products. An individual was considered to characterize fragrance sensitivity if they experienced one or more types of health problems from one or more types of fragranced products and exposure contexts (Steinemann, 2016). A “fragranced consumer product” (or “fragranced product,” for brevity) is a chemically formulated product with the addition of a fragrance or scent (Steinemann, 2015).

Fragranced product types were categorized as follows: air fresheners and deodorizers, personal care products, cleaning supplies, laundry products, household products, fragrance, and other. Specific exposure contexts were as follows: air fresheners or deodorizers used in public restrooms and other environments; scented laundry products coming from a dryer vent; being in a room after it was cleaned with scented cleaning products; being near someone wearing a fragranced product; entering a business with the scent of fragranced products; fragranced soap used in public restrooms; and ability to access environments that used fragranced products. The survey also investigated effects of fragranced product exposure in the workplace, and preferences for fragrance-free environments and policies.

Health effects were categorized as follows: migraine headaches; asthma attacks; neurological problems; respiratory problems; skin problems; cognitive problems; mucosal symptoms; immune system problems; gastrointestinal problems; cardiovascular problems; musculoskeletal problems; and other. Data on fragranced product exposures and effects were derived from a survey of the general population (Steinemann, 2017), while the present study focuses specifically on effects on the sub-populations of individuals with MCS or chemical sensitivity. (See “Survey Data”)

## 3 Results

Across Australia, 6.5% report medically diagnosed MCS, 18.9% report chemical sensitivity, and 19.9% either or both.

For co-occurrence with asthma: 74.6% of people with MCS are asthmatic; that is, diagnosed with asthma (40.8%), an asthma-like condition (47.9%), or both. Also, 56.5% of people with chemical sensitivity are asthmatic; that is, diagnosed with asthma (32.9%), an asthma-like condition (28.5%), or both. (See Table 1.)

**Table 1:** Prevalence and co-occurrence of MCS and chemical sensitivity with asthma and fragrance sensitivity.

	Gen pop	MCS diag	ChemSens	MCS/ChemSens
Total (N)	1098	71	207	218
(% relative to general population)	100.0%	6.5%	18.9%	19.9%
	N	N	N	N
	% of column total	% of column total	% of column total	% of column total
MCS diagnosed	71	71	60	71
	6.5%	100.0%	29.0%	32.6%
Chemically sensitive	207	60	207	207
	18.9%	84.5%	100.0%	95.0%
MCS diagnosed or chemically sensitive or both	218	71	207	218
	19.9%	100.0%	100.0%	100.0%
Asthma diagnosed	176	29	68	70
	16.0%	40.8%	32.9%	32.1%
Asthma-like condition diagnosed	151	34	59	64
	13.8%	47.9%	28.5%	29.4%
Asthmatic (asthma or asthma-like condition or both)	313	53	117	123
	28.5%	74.6%	56.5%	56.4%
Fragrance sensitive	362	65	171	179
	33.0%	91.5%	82.6%	82.1%

Gen Pop = general population (including sub-populations of MCS and ChemSens).

MCS Diag = medically diagnosed with MCS.

ChemSens = self-reported chemical sensitivity.

MCS/ChemSens = medically diagnosed with MCS, or self-reported chemical sensitivity, or both.

For co-occurrence with fragrance sensitivity: 91.5% of people with diagnosed MCS are also fragrance sensitive (Table 1), reporting one or more types of health problems, such as respiratory difficulties (56.3%) and migraine headaches (46.5%), when exposed to fragranced consumer products (see Table 2). Also, 82.6% of people with chemical sensitivity are also fragrance sensitive (Table 1), reporting one or more types of health problems when exposed to fragranced products (see Table 2).

**Table 2:** Health problems (frequency and type) from exposure to fragranced consumer products.

	Gen pop	MCS diag	ChemSens	MCS/ChemSens
Total (N)	1098	71	207	218
(% relative to General Population)	100.0%	6.5%	18.9%	19.9%
	(N)	(N)	(N)	(N)
	(% of column total)	(% of column total)	(% of column total)	(% of column total)
Fragrance sensitive	362	65	171	179
	33.0%	91.5%	82.6%	82.1%

Health problems from exposure to				
Air fresheners or deodorizers	180	48	106	110
	16.4%	67.6%	51.2%	50.5%
Scented laundry products from a dryer vent	67	35	48	52
	6.1%	49.3%	23.2%	23.9%
Room cleaned with scented products	168	51	115	119
	15.3%	71.8%	55.6%	54.6%
Someone wearing a fragranced product	213	47	115	117
	19.4%	66.2%	55.6%	53.7%
Any type of fragranced consumer product	223	56	132	136
	20.3%	78.9%	63.8%	62.4%

Type of health problem				
*Migraine headaches	110	33	64	68
	10.0%	46.5%	30.9%	31.2%
*Asthma attacks	83	28	52	54
	7.6%	39.4%	25.1%	24.8%
*Neurological problems (e.g., dizziness, seizures, head pain, fainting, loss of coordination)	49	19	32	34
	4.5%	26.8%	15.5%	15.6%
*Respiratory problems (e.g., difficulty breathing, coughing, shortness of breath)	183	40	98	102
	16.7%	56.3%	47.3%	46.8%
*Skin problems (e.g., rashes, hives, red skin, tingling skin, dermatitis)	104	36	67	71
	9.5%	50.7%	32.4%	32.6%
*Cognitive problems (e.g., difficulties thinking, concentrating, or remembering)	45	27	32	36
	4.1%	38.0%	15.5%	16.5%
*Mucosal symptoms (e.g., watery or red eyes, nasal congestion, sneezing)	154	34	87	90
	14.0%	47.9%	42.0%	41.3%
*Immune system problems (e.g., swollen lymph glands, fever, fatigue)	36	20	23	25
	3.3%	28.2%	11.1%	11.5%
*Gastrointestinal problems (e.g., nausea, bloating, cramping, diarrhea)	36	14	22	23
	3.3%	19.7%	10.6%	10.6%
*Cardiovascular problems (e.g., fast or irregular heartbeat, jitteriness, chest discomfort)	33	14	20	21
	3.0%	19.7%	9.7%	9.6%
*Musculoskeletal problems (e.g., muscle or joint pain, cramps, weakness)	29	15	17	19
	2.6%	21.1%	8.2%	8.7%
*Other	21	–	4	4
	1.9%	–	1.9%	1.8%

Specific fragranced product exposures that trigger health problems for people with MCS include, but are not limited to, the following: air fresheners and deodorizers (67.6%), scented laundry products coming from a dryer vent (49.3%), being in a room recently cleaned with scented products (71.8%), being near someone wearing a fragranced product (66.2%), and other types of fragranced consumer products (78.9%) (see Table 2).

Importantly, for 55.4% of people with MCS, the severity of these health problems was potentially disabling according to the criterion of the Australian Disability Discrimination Act (DDA, 1992), as assessed by a positive response to the question: “Do any of these health problems mean a total or partial loss of bodily or mental functions, for you personally?” (See “Survey Data”)

Fragranced products also restrict access in society for people with MCS: 64.8% are unable or reluctant to use public restrooms that have an air freshener, deodorizer, or scented product; 57.7% are unable or reluctant to wash hands in a public place if the soap is fragranced; 64.8% enter a business but then leave as quickly as possible due to a fragranced product; and 77.5% have been prevented from going someplace because a fragranced product would make them sick.

Significantly, 52.1% of those with MCS lost workdays or a job in the past year due to illness from fragranced product exposure in the workplace, 77.5% would support a fragrance-free policy in the workplace, and 80.3% would prefer that health care facilities and professionals were fragrance-free.

Demographic proportions of diagnosed MCS are 47.9% male and 52.1% female, compared with the general population of 49.5% male and 50.5% female. Thus, diagnosed MCS has a slight female bias (+1.6%). Relative to gender and age, the highest bias (percentage MCS greater than general population) is Male 25–34 (+8.4%).

## 4 Discussion

Results of this study indicate that MCS is widespread in the Australian population, affecting an estimated 1 million adults, with chemical sensitivity affecting another 2 million adults (ABS, 2016).

Among people diagnosed with MCS, 74.6% report being also diagnosed with asthma or an asthma-like condition. People with MCS are proportionally more likely to be asthmatic than people without MCS (prevalence odds ratio 8.7; 95% confidence interval 5.0–15.1).

In addition, among people diagnosed with MCS, 91.5% report fragrance sensitivity. People with MCS are proportionally more likely to be fragrance sensitive than people without MCS (prevalence odds ratio 26.6; 95% confidence interval 11.4–62.1).

Fragranced consumer products can trigger severe and potentially disabling health effects in a majority of people with MCS. Consequently, people with MCS are prevented from accessing restrooms, businesses, workplaces, and public places due to risks of adverse health effects from fragranced consumer products. A majority of people with MCS have lost workdays or a job, in the past year, due to exposure to fragranced products in the workplace.

In light of these results, a logical and prudent step would be to implement fragrance-free policies in workplaces and other environments, which could improve access for people with MCS and reduce potential health risks and liability. Even among the general population, most Australians would support fragrance-free workplaces, health care facilities, and health care professionals (Steinemann, 2017).

Study strengths include the following: (a) the sample population is statistically representative of age, gender, and region in Australia; (b) the 1098 respondents were randomly recruited from a large web-based panel, developed from multiple sources to reflect population characteristics; and (c) the survey used questions from a large national study in the US previously conducted and published (Steinemann, 2018). Study limitations include the following: (a) only adults (ages 18–65) were surveyed, which excludes data from other age groups; (b) the survey relied on self-reported data, although a standard and widely accepted approach for epidemiological research; (c) the cross-sectional design limits the investigation of temporal associations between exposures and effects; and (d) MCS and chemical sensitivity lack standard diagnostic criteria, although the survey replicated questions from prior large-scale studies to promote consistency and comparability.

## 5 Conclusion

MCS is a serious and potentially disabling health condition and is exacerbated by exposure to common chemically formulated products. Reducing exposure to problematic sources is critical for both primary and secondary prevention: to prevent more people from acquiring MCS, and to reduce the frequency and severity of adverse health and societal effects among an estimated 3 million Australians who already have chemical sensitivity or MCS.

The following are the supplementary data related to this article.

Supplementary material 1 Survey Methods. Supplementary material 1

Supplementary material 2 Survey Data. Supplementary material 2

## Conflicts of interest

None.
