# Supplementary material for: Prevalence and effects of multiple chemical sensitivities in Australia
Source: Prev Med Rep. 2018 Mar 10;10:191–4. doi: 10.1016/j.pmedr.2018.03.007 (PMC5984225; doi:10.1016/j.pmedr.2018.03.007)
Supplement: Supplementary material 2 — Survey Data. [file mmc2.pdf]

Table 1

Australia

Base: All Respondents

	GenPop	MCS	ChemSens	MCS/ChemSens
**Total**	1098	71	207	218
	100.0%	6.5%	18.9%	19.9%

Table 2

What is your gender?

Base: All Respondents

	GenPop	MCS	ChemSens	MCS/ChemSens
**Total**	1098	71	207	218
	100.00%	100.00%	100.00%	100.00%
**Male**	543	34	73	79
	49.50%	47.90%	35.30%	36.20%
**Female**	555	37	134	139
	50.50%	52.10%	64.70%	63.80%
**Other**	-	-	-	-
	-	-	-	-

Table 3

What is your age?

Base: All Respondents

	GenPop	MCS	ChemSens	MCS/ChemSens
**Total**	1098	71	207	218
	100.00%	100.00%	100.00%	100.00%
**18-24 (21)**	156	11	24	28
	14.20%	15.50%	11.60%	12.80%
**25-34 (29.5)**	239	20	56	57
	21.80%	28.20%	27.10%	26.10%
**35-44 (39.5)**	256	14	46	49
	23.30%	19.70%	22.20%	22.50%
**45-54 (49.5)**	241	20	47	50
	21.90%	28.20%	22.70%	22.90%
**55-65 (60)**	206	6	34	34
	18.80%	8.50%	16.40%	15.60%

Table 4

AUSTRALIA Region

Base: All Respondents

	GenPop	MCS	ChemSens	MCS/ChemSens
**Total**	1098	71	207	218
	100.00%	100.00%	100.00%	100.00%
**Australian Capital Territory**	20	3	6	7
	1.80%	4.20%	2.90%	3.20%
**New South Wales**	360	30	66	70
	32.80%	42.30%	31.90%	32.10%
**Northern Territory**	7	-	1	1
	0.60%	-	0.50%	0.50%
**Queensland**	217	12	45	47
	19.80%	16.90%	21.70%	21.60%
**South Australia**	85	6	17	17
	7.70%	8.50%	8.20%	7.80%
**Tasmania**	25	2	4	5
	2.30%	2.80%	1.90%	2.30%
**Victoria**	278	15	49	52
	25.30%	21.10%	23.70%	23.90%
**Western Australia**	106	3	19	19
	9.70%	4.20%	9.20%	8.70%

Table 5

Q2. Do you experience any health problems when exposed to air fresheners or deodorizers?

Base: All Respondents

	GenPop	MCS	ChemSens	MCS/ChemSens
**Total**	1098	71	207	218
	100.00%	100.00%	100.00%	100.00%
**Yes**	180	48	106	110
	16.40%	67.60%	51.20%	50.50%
**No**	760	17	61	65
	69.20%	23.90%	29.50%	29.80%
**Don't know/not sure**	157	6	40	43
	14.30%	8.50%	19.30%	19.70%
**Decline to answer**	1	-	-	-
	0.10%	-	-	-

Table 6

BA. Which of the following health problems do you experience?

Base: Respondents who experienced below health problems when exposed to air fresheners or deodorizers

	GenPop	MCS	ChemSens	MCS/ChemSens
**Total**	180	48	106	110
	100.00%	100.00%	100.00%	100.00%
**Migraine headaches**	46	16	28	31
	25.60%	33.30%	26.40%	28.20%
**Asthma attacks**	49	17	33	35
	27.20%	35.40%	31.10%	31.80%
**Neurological problems (e.g., dizziness, seizures,**	24	9	16	17
**head pain, fainting, loss of coordination)**	13.30%	18.80%	15.10%	15.50%
**Respiratory problems (e.g., difficulty breathing,**	100	24	62	63
**coughing, shortness of breath)**	55.60%	50.00%	58.50%	57.30%
**Skin problems (e.g., rashes, hives, red skin,**	53	22	42	44
**tingling skin, dermatitis)**	29.40%	45.80%	39.60%	40.00%
**Cognitive problems (e.g., difficulties thinking,**	21	11	15	16
**concentrating, or remembering)**	11.70%	22.90%	14.20%	14.50%
**Mucosal symptoms (e.g., watery or red eyes, nasal**	68	18	47	48
**congestion, sneezing)**	37.80%	37.50%	44.30%	43.60%
**Immune system problems (e.g., swollen lymph**	20	11	13	14
**glands, fever, fatigue)**	11.10%	22.90%	12.30%	12.70%
**Gastrointestinal problems (e.g., nausea, bloating,**	16	7	11	12
**cramping, diarrhea)**	8.90%	14.60%	10.40%	10.90%
**Cardiovascular problems (e.g., fast or irregular**	21	11	12	13
**heartbeat, jitteriness, chest discomfort)**	11.70%	22.90%	11.30%	11.80%
**Musculoskeletal problems (e.g., muscle or joint**	18	8	11	12
**pain, cramps, weakness)**	10.00%	16.70%	10.40%	10.90%
**Other**	6	-	-	-
	3.30%	-	-	-
**SUM**	442	154	290	305
	245.60%	320.80%	273.60%	277.30%

Table 7

Q3. Do you experience any health problems from the scent of laundry products coming from a dryer vent?

Base: All Respondents

	GenPop	MCS	ChemSens	MCS/ChemSens
**Total**	1098	71	207	218
	100.00%	100.00%	100.00%	100.00%
**Yes**	67	35	48	52
	6.10%	49.30%	23.20%	23.90%
**No**	909	27	117	123
	82.80%	38.00%	56.50%	56.40%
**Don't know/not sure**	120	9	42	43
	10.90%	12.70%	20.30%	19.70%
**Decline to answer**	2	-	-	-
	0.20%	-	-	-

Table 8

BA. Which of the following health problems do you experience?

Base: Respondents who experienced below health problems from the scent of laundry products coming from a dryer vent

	GenPop	MCS	ChemSens	MCS/ChemSens
**Total**	67	35	48	52
	100.00%	100.00%	100.00%	100.00%
**Migraine headaches**	14	7	11	12
	20.90%	20.00%	22.90%	23.10%
**Asthma attacks**	15	11	12	13
	22.40%	31.40%	25.00%	25.00%
**Neurological problems (e.g., dizziness, seizures,**	8	6	6	7
**head pain, fainting, loss of coordination)**	11.90%	17.10%	12.50%	13.50%
**Respiratory problems (e.g., difficulty breathing,**	28	15	20	22
**coughing, shortness of breath)**	41.80%	42.90%	41.70%	42.30%
**Skin problems (e.g., rashes, hives, red skin,**	23	14	16	19
**tingling skin, dermatitis)**	34.30%	40.00%	33.30%	36.50%
**Cognitive problems (e.g., difficulties thinking,**	11	8	9	10
**concentrating, or remembering)**	16.40%	22.90%	18.80%	19.20%
**Mucosal symptoms (e.g., watery or red eyes, nasal**	17	10	15	16
**congestion, sneezing)**	25.40%	28.60%	31.30%	30.80%
**Immune system problems (e.g., swollen lymph**	20	13	15	16
**glands, fever, fatigue)**	29.90%	37.10%	31.30%	30.80%
**Gastrointestinal problems (e.g., nausea, bloating,**	13	7	9	10
**cramping, diarrhea)**	19.40%	20.00%	18.80%	19.20%
**Cardiovascular problems (e.g., fast or irregular**	15	10	12	13
**heartbeat, jitteriness, chest discomfort)**	22.40%	28.60%	25.00%	25.00%
**Musculoskeletal problems (e.g., muscle or joint**	10	7	7	8
**pain, cramps, weakness)**	14.90%	20.00%	14.60%	15.40%
**Other**	2	-	-	-
	3.00%	-	-	-
**SUM**	176	108	132	146
	262.70%	308.60%	275.00%	280.80%

Table 9

Q4. Do you experience any health problems from being in a room after it has been cleaned with scented products?

Base: All Respondents

	GenPop	MCS	ChemSens	MCS/ChemSens
**Total**	1098	71	207	218
	100.00%	100.00%	100.00%	100.00%
**Yes**	168	51	115	119
	15.30%	71.80%	55.60%	54.60%
**No**	817	15	73	77
	74.40%	21.10%	35.30%	35.30%
**Don't know/not sure**	110	5	19	22
	10.00%	7.00%	9.20%	10.10%
**Decline to answer**	3	-	-	-
	0.30%	-	-	-

Table 10

BA. Which of the following health problems do you experience?

Base: Respondents who experienced below health problems from being in a room after it has been cleaned with scented products

	GenPop	MCS	ChemSens	MCS/ChemSens
**Total**	168	51	115	119
	100.00%	100.00%	100.00%	100.00%
**Migraine headaches**	38	13	26	28
	22.60%	25.50%	22.60%	23.50%
**Asthma attacks**	27	13	23	24
	16.10%	25.50%	20.00%	20.20%
**Neurological problems (e.g., dizziness, seizures,**	17	10	13	15
**head pain, fainting, loss of coordination)**	10.10%	19.60%	11.30%	12.60%
**Respiratory problems (e.g., difficulty breathing,**	82	27	58	61
**coughing, shortness of breath)**	48.80%	52.90%	50.40%	51.30%
**Skin problems (e.g., rashes, hives, red skin,**	31	15	21	24
**tingling skin, dermatitis)**	18.50%	29.40%	18.30%	20.20%
**Cognitive problems (e.g., difficulties thinking,**	20	13	16	18
**concentrating, or remembering)**	11.90%	25.50%	13.90%	15.10%
**Mucosal symptoms (e.g., watery or red eyes, nasal**	66	19	54	55
**congestion, sneezing)**	39.30%	37.30%	47.00%	46.20%
**Immune system problems (e.g., swollen lymph**	18	13	14	16
**glands, fever, fatigue)**	10.70%	25.50%	12.20%	13.40%
**Gastrointestinal problems (e.g., nausea, bloating,**	16	10	13	14
**cramping, diarrhea)**	9.50%	19.60%	11.30%	11.80%
**Cardiovascular problems (e.g., fast or irregular**	14	9	11	12
**heartbeat, jitteriness, chest discomfort)**	8.30%	17.60%	9.60%	10.10%
**Musculoskeletal problems (e.g., muscle or joint**	11	8	8	10
**pain, cramps, weakness)**	6.50%	15.70%	7.00%	8.40%
**Other**	7	-	2	2
	4.20%	-	1.70%	1.70%
**SUM**	347	150	259	279
	206.50%	294.10%	225.20%	234.50%

Table 11

Q5. Do you experience any health problems from being near someone who is wearing a fragranced product?

Base: All Respondents

	GenPop	MCS	ChemSens	MCS/ChemSens
**Total**	1098	71	207	218
	100.00%	100.00%	100.00%	100.00%
**Yes**	213	47	115	117
	19.40%	66.20%	55.60%	53.70%
**No**	793	18	70	76
	72.20%	25.40%	33.80%	34.90%
**Don't know/not sure**	90	5	22	24
	8.20%	7.00%	10.60%	11.00%
**Decline to answer**	2	1	-	1
	0.20%	1.40%	-	0.50%

Table 12

BA. Which of the following health problems do you experience?

Base: Respondents who experienced below health problems from being near someone who is wearing a fragranced product

	GenPop	MCS	ChemSens	MCS/ChemSens
**Total**	213	47	115	117
	100.00%	100.00%	100.00%	100.00%
**Migraine headaches**	54	14	32	33
	25.40%	29.80%	27.80%	28.20%
**Asthma attacks**	37	14	23	24
	17.40%	29.80%	20.00%	20.50%
**Neurological problems (e.g., dizziness, seizures,**	26	9	19	19
**head pain, fainting, loss of coordination)**	12.20%	19.10%	16.50%	16.20%
**Respiratory problems (e.g., difficulty breathing,**	91	22	52	53
**coughing, shortness of breath)**	42.70%	46.80%	45.20%	45.30%
**Skin problems (e.g., rashes, hives, red skin,**	19	10	13	13
**tingling skin, dermatitis)**	8.90%	21.30%	11.30%	11.10%
**Cognitive problems (e.g., difficulties thinking,**	17	10	13	14
**concentrating, or remembering)**	8.00%	21.30%	11.30%	12.00%
**Mucosal symptoms (e.g., watery or red eyes, nasal**	87	17	52	53
**congestion, sneezing)**	40.80%	36.20%	45.20%	45.30%
**Immune system problems (e.g., swollen lymph**	14	10	11	11
**glands, fever, fatigue)**	6.60%	21.30%	9.60%	9.40%
**Gastrointestinal problems (e.g., nausea, bloating,**	16	6	12	12
**cramping, diarrhea)**	7.50%	12.80%	10.40%	10.30%
**Cardiovascular problems (e.g., fast or irregular**	13	6	10	10
**heartbeat, jitteriness, chest discomfort)**	6.10%	12.80%	8.70%	8.50%
**Musculoskeletal problems (e.g., muscle or joint**	13	10	11	11
**pain, cramps, weakness)**	6.10%	21.30%	9.60%	9.40%
**Other**	9	-	2	2
	4.20%	-	1.70%	1.70%
**SUM**	396	128	250	255
	185.90%	272.30%	217.40%	217.90%

Table 13

Q6. In general, do you experience any health problems from exposure to any type of fragranced product?

Base: All Respondents

	GenPop	MCS	ChemSens	MCS/ChemSens
**Total**	1098	71	207	218
	100.00%	100.00%	100.00%	100.00%
**Yes**	223	56	132	136
	20.30%	78.90%	63.80%	62.40%
**No**	778	12	58	62
	70.90%	16.90%	28.00%	28.40%
**Don't know/not sure**	96	3	17	20
	8.70%	4.20%	8.20%	9.20%
**Decline to answer**	1	-	-	-
	0.10%	-	-	-

Table 14

BA. Which of the following health problems do you experience?

Base: Respondents who experienced below health problems from exposure to any type of fragranced product

	GenPop	MCS	ChemSens	MCS/ChemSens
**Total**	223	56	132	136
	100.00%	100.00%	100.00%	100.00%
**Migraine headaches**	51	14	33	34
	22.90%	25.00%	25.00%	25.00%
**Asthma attacks**	40	13	29	30
	17.90%	23.20%	22.00%	22.10%
**Neurological problems (e.g., dizziness, seizures,**	22	11	17	17
**head pain, fainting, loss of coordination)**	9.90%	19.60%	12.90%	12.50%
**Respiratory problems (e.g., difficulty breathing,**	110	28	71	71
**coughing, shortness of breath)**	49.30%	50.00%	53.80%	52.20%
**Skin problems (e.g., rashes, hives, red skin,**	54	21	39	39
**tingling skin, dermatitis)**	24.20%	37.50%	29.50%	28.70%
**Cognitive problems (e.g., difficulties thinking,**	19	13	16	18
**concentrating, or remembering)**	8.50%	23.20%	12.10%	13.20%
**Mucosal symptoms (e.g., watery or red eyes, nasal**	83	21	59	61
**congestion, sneezing)**	37.20%	37.50%	44.70%	44.90%
**Immune system problems (e.g., swollen lymph**	13	9	11	11
**glands, fever, fatigue)**	5.80%	16.10%	8.30%	8.10%
**Gastrointestinal problems (e.g., nausea, bloating,**	14	6	10	10
**cramping, diarrhea)**	6.30%	10.70%	7.60%	7.40%
**Cardiovascular problems (e.g., fast or irregular**	13	7	11	11
**heartbeat, jitteriness, chest discomfort)**	5.80%	12.50%	8.30%	8.10%
**Musculoskeletal problems (e.g., muscle or joint**	11	9	11	11
**pain, cramps, weakness)**	4.90%	16.10%	8.30%	8.10%
**Other**	8	-	2	2
	3.60%	-	1.50%	1.50%
**SUM**	438	152	309	315
	196.40%	271.40%	234.10%	231.60%

Table 15

Q2/Q3/Q4/Q5/Q6. People Who Answer “Yes” To One Or More Of These Questions: Q2/Q3/Q4/Q5/Q6 (fragrance sensitive group).

Base: All Respondents

	GenPop	MCS	ChemSens	MCS/ChemSens
**Total**	1098	71	207	218
	100.00%	100.00%	100.00%	100.00%
**Yes**	362	65	171	179
	33.00%	91.50%	82.60%	82.10%

Table 16

BA-Q2/Q3/Q4/Q5/Q6. People who answer “Yes” to each type of health problem under BA for each of these questions Q2/Q3/Q4/Q5/Q6 (fragrance sensitive group)

Base: All Respondents

	GenPop	MCS	ChemSens	MCS/ChemSens
**Total**	1098	71	207	218
	100.00%	100.00%	100.00%	100.00%
**Migraine headaches**	110	33	64	68
	10.00%	46.50%	30.90%	31.20%
**Asthma attacks**	83	28	52	54
	7.60%	39.40%	25.10%	24.80%
**Neurological problems (e.g., dizziness, seizures,**	49	19	32	34
**head pain, fainting, loss of coordination)**	4.50%	26.80%	15.50%	15.60%
**Respiratory problems (e.g., difficulty breathing,**	183	40	98	102
**coughing, shortness of breath)**	16.70%	56.30%	47.30%	46.80%
**Skin problems (e.g., rashes, hives, red skin,**	104	36	67	71
**tingling skin, dermatitis)**	9.50%	50.70%	32.40%	32.60%
**Cognitive problems (e.g., difficulties thinking,**	45	27	32	36
**concentrating, or remembering)**	4.10%	38.00%	15.50%	16.50%
**Mucosal symptoms (e.g., watery or red eyes, nasal**	154	34	87	90
**congestion, sneezing)**	14.00%	47.90%	42.00%	41.30%
**Immune system problems (e.g., swollen lymph**	36	20	23	25
**glands, fever, fatigue)**	3.30%	28.20%	11.10%	11.50%
**Gastrointestinal problems (e.g., nausea, bloating,**	36	14	22	23
**cramping, diarrhea)**	3.30%	19.70%	10.60%	10.60%
**Cardiovascular problems (e.g., fast or irregular**	33	14	20	21
**heartbeat, jitteriness, chest discomfort)**	3.00%	19.70%	9.70%	9.60%
**Musculoskeletal problems (e.g., muscle or joint**	29	15	17	19
**pain, cramps, weakness)**	2.60%	21.10%	8.20%	8.70%
**Other**	21	-	4	4
	1.90%	-	1.90%	1.80%

Table 17

DDA: Do any of these health problems mean a total or partial loss of bodily or mental functions, for you personally?

	GenPop	MCS	ChemSens	MCS/ChemSens
**Total**	362	65	171	179
	100.00%	100.00%	100.00%	100.00%
**Yes**	62	36	47	49
	17.10%	55.40%	27.50%	27.40%
**No**	269	23	107	111
	74.30%	35.40%	62.60%	62.00%
**Don't know/not sure**	31	6	17	19
	8.60%	9.20%	9.90%	10.60%
**Decline to answer**	-	-	-	-
	-	-	-	-

Table 18

Compared to other people, do you consider yourself allergic or unusually sensitive to everyday chemicals like those in household cleaning products, paints, perfumes, detergents, insect spray and things like that?

Base: All Respondents

	GenPop	MCS	ChemSens	MCS/ChemSens
**Total**	1098	71	207	218
(Percent of General Population)	100.0%	6.5%	18.9%	19.9%
**Yes**	207	60	207	207
	18.90%	84.50%	100.00%	95.00%
**No**	797	6	-	6
	72.60%	8.50%	-	2.80%
**Don't know/not sure**	93	5	-	5
	8.50%	7.00%	-	2.30%
**Decline to answer**	1	-	-	-
	0.10%	-	-	-

Table 19

Has a doctor or health care professional ever told you that you have multiple chemical sensitivities?

Base: All Respondents

	GenPop	MCS	ChemSens	MCS/ChemSens
**Total**	1098	71	207	218
(Percent of General Population)	100.0%	6.5%	18.9%	19.9%
**Yes**	71	71	60	71
	6.50%	100.00%	29.00%	32.60%
**No**	986	-	134	134
	89.80%	-	64.70%	61.50%
**Don't know/not sure**	40	-	13	13
	3.60%	-	6.30%	6.00%
**Decline to answer**	1	-	-	-
	0.10%	-	-	-

Table 20

Has a doctor or health care professional ever told you that you have asthma or an asthma-like condition?

Base: All Respondents

	GenPop	MCS	ChemSens	MCS/ChemSens
**Total**	1098	71	207	218
	100.00%	100.00%	100.00%	100.00%
**Yes - asthma**	176	29	68	70
	16.00%	40.80%	32.90%	32.10%
**Yes - asthma-like condition**	151	34	59	64
	13.80%	47.90%	28.50%	29.40%
**Yes - Asthma or Asthma-like condition or Both**	313	53	117	123
	28.50%	74.60%	56.50%	56.40%
**No**	740	14	85	87
	67.40%	19.70%	41.10%	39.90%
**Don't know/not sure**	43	4	5	8
	3.90%	5.60%	2.40%	3.70%
**Decline to answer**	2	-	-	-
	0.20%	-	-	-

Table 21

Have you ever been unable or reluctant to use the restrooms in a public place, because of the presence of an air freshener, deodorizer, or scented product?

Base: All Respondents

	GenPop	MCS	ChemSens	MCS/ChemSens
**Total**	1098	71	207	218
	100.00%	100.00%	100.00%	100.00%
**Yes**	127	46	80	84
	11.60%	64.80%	38.60%	38.50%
**No**	920	22	118	123
	83.80%	31.00%	57.00%	56.40%
**Neutral/not sure**	49	3	9	11
	4.50%	4.20%	4.30%	5.00%
**Decline to answer**	2	-	-	-
	0.20%	-	-	-

Table 22

Have you ever been unable or reluctant to wash your hands with soap in a public place, because you know or suspect that the soap is fragranced?

Base: All Respondents

	GenPop	MCS	ChemSens	MCS/ChemSens
**Total**	1098	71	207	218
	100.00%	100.00%	100.00%	100.00%
**Yes**	113	41	65	70
	10.30%	57.70%	31.40%	32.10%
**No**	911	23	128	133
	83.00%	32.40%	61.80%	61.00%
**Neutral/not sure**	72	7	14	15
	6.60%	9.90%	6.80%	6.90%
**Decline to answer**	2	-	-	-
	0.20%	-	-	-

Table 23

If you enter a business, and you smell air fresheners or some fragranced product, do you want to leave as quickly as possible?

Base: All Respondents

	GenPop	MCS	ChemSens	MCS/ChemSens
**Total**	1098	71	207	218
	100.00%	100.00%	100.00%	100.00%
**Yes**	183	46	104	107
	16.70%	64.80%	50.20%	49.10%
**No**	773	20	72	79
	70.40%	28.20%	34.80%	36.20%
**Neutral/not sure**	141	5	31	32
	12.80%	7.00%	15.00%	14.70%
**Decline to answer**	1	-	-	-
	0.10%	-	-	-

Table 24

Have you ever been prevented from going to some place because you would be exposed to a fragrance product that would make you sick?

Base: All Respondents

	GenPop	MCS	ChemSens	MCS/ChemSens
**Total**	1098	71	207	218
	100.00%	100.00%	100.00%	100.00%
**Yes**	165	55	98	103
	15.00%	77.50%	47.30%	47.20%
**No**	832	12	90	95
	75.80%	16.90%	43.50%	43.60%
**Don't know/not sure**	99	4	19	20
	9.00%	5.60%	9.20%	9.20%
**Decline to answer**	2	-	-	-
	0.20%	-	-	-

Table 25

Has any exposure to fragranced products in your work environment caused you to become sick, lose work days, or lose a job?

Base: All Respondents

	GenPop	MCS	ChemSens	MCS/ChemSens
**Total**	1098	71	207	218
	100.00%	100.00%	100.00%	100.00%
**Yes**	85	37	59	61
	7.70%	52.10%	28.50%	28.00%
**No**	929	28	132	139
	84.60%	39.40%	63.80%	63.80%
**Don't know/not sure**	81	5	15	17
	7.40%	7.00%	7.20%	7.80%
**Decline to answer**	3	1	1	1
	0.30%	1.40%	0.50%	0.50%

Table 26

Would you be supportive of a fragrance-free policy in the workplace?

Base: All Respondents

	GenPop	MCS	ChemSens	MCS/ChemSens
**Total**	1098	71	207	218
	100.00%	100.00%	100.00%	100.00%
**Yes**	470	55	144	149
	42.80%	77.50%	69.60%	68.30%
**No**	244	12	18	23
	22.20%	16.90%	8.70%	10.60%
**Neutral/not sure**	381	4	45	46
	34.70%	5.60%	21.70%	21.10%
**Decline to answer**	3	-	-	-
	0.30%	-	-	-

Table 27

Would you prefer that health care facilities and health care professionals be fragrance-free?

Base: All Respondents

	GenPop	MCS	ChemSens	MCS/ChemSens
**Total**	1098	71	207	218
	100.00%	100.00%	100.00%	100.00%
**Yes**	474	57	142	149
	43.20%	80.30%	68.60%	68.30%
**No**	277	9	27	30
	25.20%	12.70%	13.00%	13.80%
**Neutral/not sure**	342	5	37	38
	31.10%	7.00%	17.90%	17.40%
**Decline to answer**	5	-	1	1
	0.50%	-	0.50%	0.50%

Table 28

Demographics.

Base: All Respondents

	GenPop	MCS	ChemSens	MCS/ChemSens
**Total**	1098	71	207	218
	100.00%	100.00%	100.00%	100.00%
**Male/Female**				
**All Males**	543	34	73	79
	49.50%	47.90%	35.30%	36.20%
**All Females**	555	37	134	139
	50.50%	52.10%	64.70%	63.80%
**Gender vs Age**				
**Male 18-24**	70	5	8	10
	6.40%	7.00%	3.90%	4.60%
**Male 25-34**	109	13	27	28
	9.90%	18.30%	13.00%	12.80%
**Male 35-44**	119	7	12	15
	10.80%	9.90%	5.80%	6.90%
**Male 45-54**	126	7	17	17
	11.50%	9.90%	8.20%	7.80%
**Male 55-65**	119	2	9	9
	10.80%	2.80%	4.30%	4.10%
**Female 18-24**	86	6	16	18
	7.80%	8.50%	7.70%	8.30%
**Female 25-34**	130	7	29	29
	11.80%	9.90%	14.00%	13.30%
**Female 35-44**	137	7	34	34
	12.50%	9.90%	16.40%	15.60%
**Female 45-54**	115	13	30	33
	10.50%	18.30%	14.50%	15.10%
**Female 55-65**	87	4	25	25
	7.90%	5.60%	12.10%	11.50%
